# Mass Vaccination Campaign Following Community Outbreak of Meningococcal Disease

**DOI:** 10.3201/eid0812.010421

**Published:** 2002-12

**Authors:** Gérard Krause, Carina Blackmore, Steven Wiersma, Cheryll Lesneski, Laurey Gauch, Richard S. Hopkins

**Affiliations:** * Centers for Disease Control and Prevention, Atlanta, Georgia, USA; † Florida Department of Health, Tallahassee, Florida, USA; ‡ Putnam County Health Department, Palatka, Florida, USA

**Keywords:** meningococcal vaccine, meningococcal meningitis, serotype C, bioterrorism, disease outbreaks, epidemiology

## Abstract

During December 12–29, 1998, seven patients ages 2–18 years were diagnosed with serogroup C meningococcal disease in two neighboring Florida towns with 33,000 residents. We evaluated a mass vaccination campaign implemented to control the outbreak. We maintained vaccination logs and recorded the resources used in the campaign that targeted 2- to 22-year-old residents of the two towns. A total of 13,148 persons received the vaccinations in 3 days. Vaccination coverage in the target population was estimated to be 86% to 99%. Five additional cases of serogroup C meningococcal disease occurred in the community during the year after the campaign began, four in patients who had not received the vaccine. The cost of control efforts was approximately $370,000. Although cases continued to occur, the vaccination campaign appeared to control the outbreak. Rapid implementation, a targeted approach, and high coverage were important to the campaign's success.

*Neisseria meningitidis* is a leading cause of bacterial meningitis and sepsis in children and young adults in the United States (1,2). An estimated 2,600 cases occur each year, most of them sporadic (2). Between 10 and 15 outbreaks of meningococcal disease are reported in the country annually (1,3). Outbreaks can occur in institutions as well as in communities. Communitywide outbreaks can persist for several months, and controlling them remains a major challenge in public health (4–6).

The primary method for preventing sporadic meningococcal disease is chemoprophylaxis of close contacts after a case is identified (1,7,8). However, the protective effect of chemoprophylaxis is of limited duration (6,9,10).

A quadrivalent polysaccharide meningococcal vaccine effective against *N. meningitidis* serogroup A, C, W135, and Y is available in the United States (7,11). Serogroup C *N. meningitidis* accounts for most U.S. outbreaks (3). The Advisory Committee on Immunization Practices (ACIP) has released recommendations for the use of meningococcal vaccine to control outbreaks of serogroup C meningococcal disease (11).

Identifying the need for a vaccination campaign, defining the target population, implementing the campaign rapidly, and achieving high vaccination coverage are difficult (5). Mass vaccination campaigns require major logistic efforts and often take place in an atmosphere of public anxiety (5,6,12,13). Few local and state health departments have much experience in responding to such outbreaks. Mass vaccination campaigns in response to meningococcal disease outbreaks have been reported before, but only limited information is available on the operational aspects of such efforts (4,5,9,14).

In December 1998, two neighboring towns with a combined population of 33,000 persons in Putnam County, Florida, had a community outbreak of meningococcal disease (1,3). The health department administered chemoprophylaxis to close contacts of the case-patients and investigated links between patients. Detailed results of the investigation have been described elsewhere (15). On December 29, 1998, the decision was made to implement a mass vaccination campaign to control the outbreak in the community, based on ACIP recommendations (7). We describe the epidemiology of the outbreak and the methods used for providing chemoprophylaxis and implementing the vaccination campaign. We also evaluate the results and the cost of the control efforts.

## Materials and Methods

### Case Definition

A case-patient was defined as a person in Putnam County with onset of clinically compatible illness after November 1998 and isolation of serogroup C *N. meningitidis* obtained from a normally sterile site or detection of serogroup C meningococcal polysaccharide antigen in the cerebrospinal fluid or serum (7). Pulsed-field gel electrophoresis (PFGE) was performed on culture-confirmed isolates in the state health department laboratory by using previously described methods (16,17).

### Chemoprophylaxis

Chemoprophylaxis—rifampin, ciprofloxacin, or ceftriaxone, as recommended by ACIP (7)—was given to close contacts. A close contact was defined as a household member, day-care center contact, or anyone directly exposed to the patient’s oral secretion (7,18). We documented all chemoprophylaxis provided by the health department for the first nine patients and retrospectively verified whether the persons who had received prophylaxis met the definition of a close contact.

### Vaccination Campaign

The decision to implement the vaccination campaign was based on ACIP guidelines for community-based outbreaks (7). We defined the population at risk as the population of the two neighboring towns since this area represented the smallest geographically contiguous population that included all case-patients. The target population for the vaccination campaign was defined as all residents ages 2–22 years.

The vaccination campaign took place December 30–31, 1998, and January 2, 1999. Additional vaccinations were offered at the health department by appointment until January 20, 1999, for those who belonged to the target group but did not receive the vaccine during the 3-day campaign.

We organized a single-site immunization clinic in the high school cafeteria. We used Menommune quadrivalent polysaccharide meningococcal vaccine (Aventis Pasteur [formerly Pasteur Merieux Connaught], Swiftwater, PA) in 50-dose vials originally designed for jet injector use. All adult vaccinees signed a consent form before being vaccinated. A parental consent was required for vaccinees <18 years of age.

### Public Information

Staff of the county health department, the state health department, and the school administration received question-and-answer sheets to respond to telephone inquiries from the public. The state health department installed a toll-free phone number with automated information on the vaccination campaign and posted additional information on the Internet. Health department staff held daily press conferences from December 30, 1998, through January 2, 1999.

### Vaccination Coverage

We maintained vaccination logs during the campaign, in which we documented age, sex, and address of the vaccinee as well as date of vaccination. To determine the reliability of the address information in the log, we selected a random sample from the vaccinees listed in the log. We validated the address information with the school enrollment database of the county. Only addresses of persons identified in both databases were compared. The size of the population at risk and the target population was determined by using 1998 official Florida population figures provided by the Bureau of Economic and Business Research at the University of Florida.

### Cost of the Intervention

We recorded expenditures for health department staff time and material resources associated with the efforts to control the outbreak. We did not include salaries for police and fire department personnel, local health-care providers, or the value of time and services donated by volunteers.

## Results

### Outbreak Investigation

During December 14–26, 1998, four female and three male case-patients of serogroup C meningococcal disease were reported to the health department of Putnam County (Table 1). Their ages ranged from 2 to 18 years (median 12). Two of the patients had close contact with each other so that the primary disease attack rate was 15 per 100,000. The county had received an average of one report of meningococcal disease per year in 1988–1998.

**Table 1:** Cases of serogroup C meningococcal disease, Putnam County, Florida, 1998–1999^a^

Onset date	Sex	Age	Resident of target area	Received chemoprophylaxis	Received vaccine	PFGE	Outcome
12/12/98	F	2 yrs	Yes	No	No	No isolate	No sequelae
12/12/98	F	18 yrs	Yes	No	No	Identical	No sequelae
12/18/98	M	12 yrs	Yes	No	No	Identical	No sequelae
12/25/98	F	2 yrs	Yes	No	No	Identical	No sequelae
12/25/98	F	4 yrs	Yes	No	No	Identical	No sequelae
12/26/98	M	11 yrs	Yes	No	No	Identical	No sequelae
12/26/98	M	12 yrs	Yes	No	No	No isolate	No sequelae
12/29/98	M	18 yrs	Yes	No	No	Identical	Died
01/03/99	M	25 yrs	Yes	Yes	No	Identical	No sequelae
3/25/99	M	17 yrs	No	No	No	Identical	No sequelae
5/12/99	F	6 yrs	Yes	Yes	Yes	3 bands’ difference	Necrosis of ear lobe
12/28/99	F	3 mo	Yes	No	No	Identical	Died

^a^PFGE, pulsed-field gel electrophoresis; M, male; F, female.

One case occurred the night before the campaign started. After the campaign began, four additional cases (two female, two male) of serogroup C meningococcal disease occurred until December 1999; ages of patients ranged from 3 months to 25 years (Table 1). One of the patients with onset in January 1999 had received chemoprophylaxis. Another patient, whose onset was in May, had received both chemoprophylaxis and vaccine. The other postcampaign patients had not received vaccine. PFGE performed on isolates from 10 of 12 patients showed identical patterns for the first eight isolates. The isolates from the three case-patients in March, May, and December 1999 differed by three or fewer bands from the earlier outbreak strains (Table 1).

### Chemoprophylaxis

The health department gave chemoprophylaxis to a total of 484 contacts, ranging from 7 to 108 contacts per patient. Three hundred six (63%) of those who received chemoprophylaxis were considered close contacts according to ACIP criteria (7).

### Vaccination Campaign

Law enforcement personnel from the local sheriff’s department provided crowd and traffic control. Volunteers and clerical staff served as welcome staff, handed out information sheets, and guided incoming people to the lines. The main waiting line outside the school was divided into 6–18 parallel lines inside the cafeteria. One check-in station existed for each line. Each such station was staffed with one to three clerks, who determined eligibility for persons by age and residency requirements (self-reported). Two physicians supervised the process. Vaccinees received vaccination cards and were directed to continue to one of the 6–18 parallel vaccination posts, located behind the check-in station. These posts were equipped with skin disinfectants, Band-Aids, sharps containers, and coolers with prefilled vaccine syringes. Two to four registered nurses staffed each vaccination post. A registered nurse and a physician supervised the vaccinations. Registered nurses prepared single-dose syringes in the cafeteria kitchen, adjacent to the vaccination clinic. Three to 10 persons were involved continuously in preparing the syringes. Syringes were filled from the multidose vial, capped with new needles, and stored in coolers. In a separate room adjacent to the vaccination posts, the local fire department provided staff and equipment for first aid and advanced life support. Two vaccinees needed first aid because of minor injuries, occurring as a result of syncope before or shortly after vaccination. No allergic reactions or injection site infections were reported during the campaign or afterwards. One needlestick injury occurred in a health-care worker.

### Public Information

The state health department, county health department, and school administration had difficulty coordinating the release of information to the media and the public. Some elected officials did not accept the targeted approach of the vaccination campaign. Public anxiety resulting in part from inconsistent messages and disagreements among officials hampered efforts to conduct the vaccination campaign as originally planned. The toll-free information phone line registered over 5,000 calls in 7 days. In addition to the affected county health department, health departments from neighboring counties also received hundreds of phone calls each day during the outbreak.

### Vaccination Coverage

The target population of 2- to 22-year-old residents of the two neighboring towns was 10,132. A total of 13,535 persons received the vaccine. Of these, 13,148 (97%) were vaccinated during the first 3 days of the campaign; the remaining 387 (3%) were vaccinated by appointment at the health department on January 5–20, 1999. Between 300 and 1,100 vaccinations were given per hour. During maximum workload, 1,100 vaccinations were delivered per hour by approximately 78 workers who staffed the 18 lines. According to the vaccination logs, among the 13,148 persons who received vaccine during the first 3 days, 10,076 (77%) belonged to the target population, 3,065 (23%) lived outside the target area, and 7 (<1%) were older than the target age group (Tables 2 and 3). On the basis of these numbers, vaccination coverage was 99% (10,076/10,132). Vaccination coverage was lower in the age groups 15–20 and 21–22 years than in younger children (Table 3). More than half of all persons in the target group received vaccine on day 1 of the mass campaign; 84% were immunized by the end of day 2 and 96% after day 3 (Table 2).

**Table 2:** Vaccinations by day and target area, Putnam County, Florida, 1998–1999

Vaccination dates	Vaccinees within target area	Vaccinees outside target area	Total vaccinees	% of all vaccinees in target area
12/30/98	5,398	229	5,627	96
12/31/98	3,393	656	4,049	84
1/2/99	1,292	2,180	3,472	37
Total first 3 days	10,083	3,065	13,148	77
1/2–20/99	363	24	387	94
Total	10,446	3,089	13,535	77

**Table 3:** Size of target population and number vaccinated by age group, Putnam County, Florida, 1998–1999

Age group	Estimated target population	No. (%) of target population vaccinated^a^
Approximately 2–4 yrs	1,439	1,435 (100)
5–9 yrs	2,460	2,741 (111)
10–14 yrs	2,513	2,606 (104)
15–20 yrs	2,424	2,288 (94)
21–22 yrs	1,296	1,006 (78)
Total	10,132	10,076 (99)

^a^Figures >100% result from the fact that people outside the target population received vaccination.

Among those ages 5–17 with zip codes within the target area (6,699), a sample of 191 (3%) vaccination records were selected, and the names and addresses were compared to the school enrollment database of the county. Among these, 165 (86%) matched in name and birthdate with records in the school enrollment database. Of these records, 142 (86%) had addresses within the target area in the school enrollment database, while 23 (14%, 95% confidence interval 9% to 19%) had addresses outside the target area. If 14% of the addresses for the 10,076 vaccinees with addresses within the target area were incorrect, approximately 8,664 vaccinees actually were from the target area. The overall estimate for the vaccination coverage for the first 3 days of the campaign would therefore have been 86% (8,664/10,132).

### Cost of Intervention

The cost of the public health response to the outbreak amounted to approximately $370,000; 65% of this amount was for the purchase and delivery of vaccine, 20% for personnel costs to administer it, 6% for personnel cost involved in public information, 5% for personnel cost for contact investigation and chemoprophylaxis, and 4% for other expenses (Table 4). The cost for the vaccination campaign alone totaled approximately $329,300 (vaccine, personnel for vaccine administration, and other expenses), resulting in a cost per vaccination of approximately $24.

**Table 4:** Cost of public health intervention to control meningococcal outbreak, Putnam County, Florida, 1998–1999^a^

Expenditure	Estimated working hrs^b^	Approximate cost (U.S.$)	% of total cost of intervention
Purchase and delivery of the vaccine	N/A	240,500	65
Personnel costs			
Vaccine administration	4,000	74,000	20
Public information	1,200	22,200	6
Contact investigation	1,000	18,500	5
Other	N/A	14,800	4
Total	6,200	370,000	100

^a^NA, not applicable. ^b^Average cost per hour of a public health employee was estimated to be $18.50.

## Discussion

Even accounting for incorrect addresses in the vaccination registers, the vaccination coverage in this campaign was high. Whether the vaccination campaign controlled the outbreak is difficult to prove. The five cases that occurred after the campaign had ended indicate that the population was under continuing exposure to the outbreak strain during the next year. Nevertheless, this low number of subsequent cases suggests that the vaccination campaign probably prevented additional cases that could not have been prevented by chemoprophylaxis alone (19–28). One of these cases was attributed to vaccine failure. In other studies, vaccine efficacy has been estimated to be approximately 85% (4). The size of the unvaccinated population and the number of cases were too small to determine the efficacy of vaccination in this campaign.

The outbreak response was organized within 24 hours after the ACIP criteria were fulfilled and approximately 2 weeks after the first case was reported. This response time compares favorably to that in other vaccination campaigns in response to community-based meningococcal disease outbreaks (3–5,10,14,29). Most mass vaccination campaigns designed in response to community-based outbreaks tend to last more than several weeks or months, which makes a comparison to our results difficult (5,10,29). In England a mass vaccination campaign conducted in two communities lasted approximately 1 week; 8,320 and 7,660 vaccinations were given in each community, respectively (14). In a meningococcal disease outbreak in Illinois, 125–334 vaccinations were given per hour with jet injectors; however, details were not described (30). Compared with similar reports, providing over 13,000 vaccinations in 3 days and up to 1,100 vaccinations per hour indicates that the intervention was an operational success (5,10,30).

We targeted the vaccination campaign at 2- to 22-year-olds because the vaccine is not effective in children <2 years and because at the time the decision was made none of the patients was >18 years old. We extended the upper age limit to 22 years to include students of the local college since meningococcal disease outbreaks frequently occur in college settings. While the community seemed to accept the upper and lower age limits for the target population, it did not comply well with the geographic restriction. In retrospect, a more targeted approach might be appropriate. However, enforcing a more focused vaccination strategy can be extremely difficult if interventions are needed immediately and under extreme public pressure and fear. The proportion of vaccinees from outside the target area increased each day. Comparison with the school enrollment database showed that 14% of vaccinees had addresses outside the target area. While recent address changes may have been a factor, most of these likely claimed to live in the target area to be eligible for the vaccine.

Public pressure fueled in part by conflicting information released by elected officials became so great that by the afternoon of the 2nd vaccination day restricting vaccination to the target population was impossible. As a result, on the 3rd day most people who were vaccinated lived outside the target area. In an outbreak described by Irwin et al., restricting the campaign to the target population was also difficult (14). Limiting the intervention to only 3 days probably helped to prevent mistargeting of resources, since additional vaccination days would mainly have increased the number of vaccinees from outside the target area.

The effectiveness of chemoprophylaxis in outbreak control has been questioned in previous meningococcal disease outbreaks in Canada and the United Kingdom (9,10). One problem with chemoprophylaxis is that it is effective for a limited period of time. Current recommendations provide guidance for who should receive chemoprophylaxis, but these guidelines can be difficult to implement (7,8,18). Various studies have shown that household contacts are at greater risk of acquiring meningococcal disease and would benefit most from chemoprophylaxis (18,21,22). However, the available evidence does not always indicate who else should receive chemoprophylaxis (6,18,21–28). Twenty-five percent of those who received chemoprophylaxis in this outbreak were not close contacts according to the ACIP definition (7); however, chemoprophylaxis, especially in highly publicized cases, is often given to people who do not fit the definition of a close contact (9,18,19).

The automated toll-free information hot line proved to be an efficient way to respond to the large number of calls from concerned citizens. Confusion might have been avoided if all telephone requests had been directed to only one location sufficiently equipped with phone lines and personnel. We did not brief some of the elected officials early enough to ensure consistent release of information to the public. However, the major problem was the failure of some community leaders to support the targeted approach for vaccination. Similar situations have been described with other vaccination campaigns (12,13,29). The importance of having a close, ongoing relationship between public health and other community leaders cannot be overemphasized. When public health officials have the trust, confidence, and appreciation of other leaders, communicating a unified message is more likely. The circumstances of this outbreak were similar to those expected in the event of a bioterrorist attack, where rapid intervention is required, and the lessons from this outbreak response will be used in Florida to plan for such an event.

The public health response to this outbreak involved considerable expense. In addition to the costs to the health department for personnel and material, the contributions of other state and county agencies, as well as individual volunteers, were substantial. During a school-based vaccination campaign against meningococcal disease in Illinois, approximately $22.50 was spent per vaccination, which is close to our estimate (30). However, during a mass vaccination campaign in the state of Washington, the cost for vaccine administration alone was estimated to be $20 per vaccinee, not including the cost of the vaccine (29). Estimating the cost of future interventions of this type will need to take into account the fact that 50-dose vaccine vials, such as used in this campaign, are no longer available in the United States. The difference in cost is substantial; if we had used 10-dose vials, the cost for the vaccine alone would have increased by approximately 80% to $440,000. This additional cost would have resulted in a cost per vaccinee of $39.

Our decision to implement a vaccination campaign followed ACIP criteria based on epidemiologic and laboratory information. Keeping the public, elected officials, health-care providers, neighboring health departments, and the media informed should be a priority. The vaccination campaign should be confined to the target population to ensure that high vaccination coverage is accomplished early among those most at risk. Based on our experience, providing vaccinations at a single site and in a short time period to maintain control over the operation and conserve resources seems preferable.
